# Lifestyle medicine pillars as predictors of psychological flourishing

**DOI:** 10.3389/fpsyg.2022.963806

**Published:** 2022-07-29

**Authors:** Jolanta Burke, Pádraic James Dunne

**Affiliations:** Royal College of Surgeons in Ireland, University of Medicine and Health Sciences, Dublin, Ireland

**Keywords:** lifestyle medicine, PERMA model, mental health continuum model, physical health and psychological wellbeing, flourishing, positive psychology

## Abstract

Positive Psychology Interventions (PPI) are widely applied to improving wellbeing and helping individuals flourish. At the same time, Lifestyle Medicine (LM) offers an opportunity to boost PPI and psychological research, by expanding its capacity beyond psychology, to include the body and social environment. However, little is known about the relationship between LM and positive psychology flourishing models. Flourishing is as a stage of optimal human functioning that goes beyond moderate wellbeing. The objective of this cross-sectional study was to, (1) identify which of the six LM pillars (sleep, physical exercise, eating well, alcohol intake, social engagement, stress management) best-predicted flourishing; (2) examine the relationship between the number of LM pillars used by individuals and flourishing; and (3) determine the odds of using LM pillars by flourishers. A total of 1,112 participants, mostly female professionals (73%), aged 40–59 (77%), based in Ireland, completed an online survey. Regression analysis showed that all six LM pillars predicted flourishing as measured by the PERMA Profiler (including the Physical Health component) and the Mental Health Continuum (MHC). Moreover, the chi-square and odds ratio analysis showed that those who flourished were three times more likely to use 3–6 LM pillars than those who were moderately well; and nine times more likely than languishers. The results are discussed in the context of their contribution to enhancing the population’s health and wellbeing.

## Introduction

Since its inception, positive psychology aimed to explore valued subjective experiences, such as wellbeing (Seligman and Csikszentmihalyi, 2000), which was later renamed flourishing. Flourishing is as a stage of optimal human functioning that goes beyond moderate wellbeing. There are two main models and assessments of flourishing: the Wellbeing Model, otherwise known as the PERMA model (Seligman, 2013), and the Mental Health Continuum (MHC) Model (Keyes, 2002). While other wellbeing models, such as Psychological Wellbeing (Ryff, 1989), or Subjective Wellbeing (Diener et al., 2018) focus primarily on either affective or cognitive aspects of wellbeing, flourishing models incorporate both. Furthermore, what differentiates flourishing models from other wellbeing frameworks is that they are related to the positive assets that prevent mental illness and improve wellbeing, they combine affective and cognitive components, and they assume that wellbeing is a composite of all the elements comprising the model (Moneta, 2014; Burke and Arslan, 2021). However, more research is required to assess whether the assumptions relating to flourishing models are accurate (Ryff, 2022).

Over the years, a range of Positive Psychology Interventions (PPIs) were developed, that focused on enhancing the positive components of flourishing. These interventions provided evidence that each tool reduces mental health issues and results in positive outcomes, such as wellbeing and associated elements (Parks and Biswas-Diener, 2013). These tools are aimed at altering cognition or affect and include such interventions as gratitude, savoring, and the best-possible-self activities (Sin and Lyubomirsky, 2009; White et al., 2019). The flourishing components of many PPIs also include happiness, relationships and locus of control (Lyubomirsky et al., 2005; Bryant and Veroff, 2007; Round and Burke, 2018; Borelli et al., 2020).

Focused on psychological tools, for years, the field of positive psychology has notoriously disregarded the body (Hefferon, 2016; Burke and Arslan, 2021). However, in recent years, calls to incorporate body and mind have paved the way toward bringing together the fields of positive psychology and lifestyle medicine (LM) (Lianov et al., 2019; Dunne and Schubert, 2021).

Lifestyle medicine involves the use of evidence-based, lifestyle and therapeutic interventions to prevent, treat and reverse non-communicable diseases (NCDs) (Frates et al., 2021). These illnesses include cardiovascular disease (CVD), respiratory disease, Type 2 Diabetes, metabolic syndrome (associated with abdominal obesity, high blood pressure, high fasting blood glucose, and triglycerides as well as abnormal blood cholesterol), certain cancers, mental health issues, and suicide (ACLM, 2022b).

Lifestyle medicine was developed to combat the emerging crisis related to the rise of NCDs, which account for approximately 41 million deaths (71%) globally each year (WHO, 2021). Eighty-five percent of the 15 million premature deaths that happen annually occur in low to middle-income countries (WHO, 2021). The specific causes of these premature deaths include CVD (17.9 million), cancer (9.3 million), respiratory illness (4.1 million), and diabetes (1.5 million) (WHO, 2021). NCD-related premature deaths in Ireland (2019) are attributed to CVD (28.7%), cancer (30.3%), respiratory disease (13.4%), mental disorders (6.3%), and diseases of the digestive system (3.5%) (CSO, 2019).

The risk factors associated with NCDs and premature death are divided into (a) genetic (predominantly of epigenetic origin), (b) environmental (air and water pollution, as well as high UV radiation from the sun), (c) sociodemographic (age, gender, race, ethnicity, general education level, health education literacy, and income), (d) the capacity to self-manage (tobacco and alcohol use, physical activity, weight, food choice, and dental care) and (e) factors related to medical conditions (medications, blood pressure, blood lipids, blood glucose, obesity, and stress) (Budreviciute et al., 2020).

The six pillars of LM include, (1) the consumption of a predominantly whole food, plant-based diet; (2) daily physical activity; (3) adequate sleep; (4) cultivating stress management techniques and behaviors; (5) avoiding risky substances, especially tobacco and alcohol; and (6) social engagement (Frates et al., 2021).

LM recognizes the importance of psychosocial wellbeing, motivation, behavior change science, readiness to change, and the therapeutic alliance between healthcare professionals and service users as significant contributors to a total state of health. Significant interest lies in integrating evidence-based psychosocial interventions such as PPI, with positive lifestyle changes related to the pillars of LM (Morton, 2018; Lianov et al., 2019; Burke and Arslan, 2021; Dunne and Schubert, 2021). However, more research is required to understand the nature and impact of integrating these approaches for better health. Recently, Przybylko et al. (2021) tested a 10-week intervention that combined PP and LM, which resulted in increased levels of flourishing in the experimental group compared with the control group, thus suggesting that LM and PPI can complement each other to enhance positive outcomes (Przybylko et al., 2021). Briefly, 510 participants were recruited into a randomized controlled trial (RCT) designed to test the capacity of a lifestyle intervention, to increase flourishing, compared to a wait-list control group. This 10-week online programme combined PPI approaches (practicing daily positive speech and developing a positive outlook, mindfulness and cultivating kindness for self and others, and exposure to nature) with LM-related practices (eating well, adequate rest and sleep, and daily exercise). Flourishing was measured using the Huppert and So conceptualization (Huppert and So, 2013).

Individual LM pillars can also have a positive impact on wellbeing and mental health. Recently, physical activity was associated with higher wellbeing and better sleep, after 3,323 Iranian citizens were surveyed during community lockdowns throughout the COVID-19 pandemic (Akbari et al., 2021). In addition, a study of more than 80,000 United Kingdom citizens found that eating fruit and vegetables daily, was linked to a significant increase in wellbeing; wellbeing peaked with 7 daily portions of fruit and vegetables (Blanchflower et al., 2013).

Recently, Burke et al. (2022) reviewed PP and LM interventions and reported over 100 tools that demonstrated positive psychological outcomes, such as flourishing for typical positive-psychological interventions. Therefore, preliminary evidence suggests that PPI and practices can likely synergize health outcomes when integrated with LM approaches and vice versa. However, more evidence is required about the relationship between LM pillars and flourishing.

The aim of this study was to examine the following questions: (1) which of the six LM pillars predicted wellbeing; (2) what is the relationship between the number of LM pillars used and flourishing; and (3) what are the odds of flourishers using LM pillars compared with participants who languished.

## Materials and methods

This is a cross-sectional study that uses data collected online between December 2020 and February 2021, the aim of which was to assess participants’ wellbeing. The current analysis explored one aspect of the study, i.e., the relationship between the two main flourishing scales the MCC and PERMA Profiler (incl. Physical Health component) with individual LM pillars. This research received ethical approval from the Social Research Ethics Committee at Maynooth University.

### Participants

One thousand, one hundred and twelve participants (73% female), located in the Republic of Ireland, completed an online survey. Forty-eight percent were over 50 years of age (*n* = 498), 36% were aged 40–49 years (*n* = 396), while 16% were in the 18 to 39 age bracket (*n* = 178). A purposive sampling method was applied in primary and post primary schools. The inclusion criteria for participation were that participants were over 18 and worked as educational leaders in a school in Ireland. Thus, all the participants were professionals employed by the Department of Education in Ireland. The survey link was sent out *via* social network and educational organizations (Irish Primary Principals Network and National Association of Principals and Deputy Principals) that agreed to disseminate it to their members.

### Measures

#### Lifestyle medicine pillars

Due to the lack of a validated instrument to assess the use of LM pillars, in the current survey, participants were asked a question “*What actions do you take to look after your physical and mental wellbeing? Please tick all that apply*.” and requested to tick all the responses that applied to them, thus participants’ responses were binary. Their options included: (1) ensuring good sleep hygiene (consistent bedtime, sufficient duration, comfort, darkness, avoid devices emitting blue light, etc.), (2) regular exercise, (3) social activities, hobbies, and interests, (4) healthful food choices most of the time, and (5) moderate alcohol intake. Each option represented the main LM pillars and the prevalent or generic behaviors associated with them. Alcohol was identified as an example of the pillar relating to substance use. In Ireland, one in five adults smoke (Sheridan et al., 2018), while seven out of ten adults consume alcohol regularly (O’Dwyer et al., 2021). Furthermore, Ireland was one of two European countries where alcohol consumption did not decline during the COVID-19 pandemic (Kilian et al., 2021). Therefore, alcohol consumption was selected as the example of substance use in the current study. To identify perceived stress, participants were asked to assess how much stress they experienced in the past 3–4 months, using a 3-point scale (*a little*; *moderate*; *a lot*). The scale was transformed into a binary variable (*a lot of stress*; *moderate*; *a little stress*) to match other variables and conduct the chi-square and the odds analysis. A 3-point scale was used because if binary “stress” vs. “no-stress” options were applied, participants who experience moderate levels of stress would self-identify as being stressed. This would skew the results, given that moderate levels of stress can be good for wellbeing (Aschbacher et al., 2013).

#### Mental health continuum

Mental health continuum is a 14-item measure on a 6-point Likert scale ranging from “never” to “every day.” It comprises three components, (1) emotional, (2) social, and (3) psychological wellbeing. The following is a sample question, “In the past month, how often did you feel … satisfied with life.” The scale was scored using Keyes’s (2009) recommended syntax as continuous data (overall MHC, and its components) and categorical data (flourishing vs. not flourishing; flourishing, moderate health, languishing). Flourishing mental health is reported when individuals experience at least one of the three signs of emotional wellbeing and at least six of the eleven signs of social and psychological wellbeing during the past month “every day” or “almost every day.” Individuals who score “never” or “once or twice” during the past month, on at least one measure of emotional wellbeing, and low levels on at least six measures of social and psychological wellbeing are reported as languishing (poor mental health). Individuals who are neither flourishing nor languishing are diagnosed with moderate mental health. Past research showed excellent reliability at *a* = 0.80 (Keyes, 2009). The reliability in the current sample was also excellent at α = 0.93.

#### PERMA profiler

The PERMA Profiler is a 23-item measure on an 11-point Likert scale. The scale responses range, from “never” to “always,” “not at all” to “completely,” or “terrible” to “excellent.” The PERMA overall score is a mean of five components (Positive Emotions, Engagement, Relationships, Meaning in Life, Accomplishment) and additional one-item question relating to happiness. Each of the five components comprises three-item questions. Question example, “Taking all things together, how happy would you say you are?” In addition to the measure of PERMA wellbeing, additional components have been added by researchers to use if required, i.e., three-item components assessing Physical Health and Negative Emotions, and one-item instrument assessing loneliness are offered as optional measures. In the current research, we added the Physical Health component to compliment the measure of psychological wellbeing, Past reliability of the scale is very good and ranges between α = 0.72–0.94 (Butler and Kern, 2016). The current study showed excellent reliability of the overall construct at α = 0.91 and the Physical Health component at α = 0.92.

### Statistical analysis

SPSS (Version 27, IBM, 2021) was used to conduct the statistical tests. The analysis included descriptive analysis, Pearson Correlation, Multiple Regression, Chi-square Test of Independence. There was no missing data, as all responses were forced.

## Results

The LM pillar most frequently used by participants to enhance their wellbeing was exercise (*n* = 680, 61.2%), followed by eating well (*n* = 650, *n* = 58.5%), sleeping (*n* = 633, 56.9%), moderating alcohol use (*n* = 572, 51.4%), social engagement (*n* = 434, 38%), and stress management (*n* = 295, 26.5%). See Table 1 for further detail. In relation to the MHC measure, most of participants were flourishing (*n* = 598, 54%), followed by those who reported being moderately well (*n* = 477, 43%), and languishing (*n* = 37, 3.3%). Furthermore, in the current sample, the mean for MHC was *M* = 3.14, SD = 0.84; for PERMA, *M* = 7.05, SD = 1.69; for the Physical Health component of PERMA, *M* = 6.95, SD = 2.37.

**TABLE 1:** Participants’ prevalence of engaging with lifestyle medicine (LM) pillars.

Lifestyle medicine pillars	*N*	%
Sleep hygiene	633	56.9
Regular exercise	680	61.2
Social activities	434	39
Healthful food choices	650	58.5
Moderate alcohol intake	572	51.4
Perceived stress	295	26.5

### Relationship between flourishing and lifestyle medicine pillars

The Pearson Correlation test was used to assess the strength of relationship between all the wellbeing measures (MHC, PERMA and Physical Health) and the six pillars of LM. Small to moderate relationship was found between all the variables at *p* < 0.001. See Table 2 for details.

**TABLE 2:** Pearson correlation between wellbeing measures and the six pillars of lifestyle medicine (LM).

	MHC	PERMA	Health	Sleep	Exercise	Social	Eating	Alcohol
PERMA	0.699*							
Health	0.466*	0.520*						
Sleep	0.217*	0.196*	0.180*					
Exercise	0.240*	0.206*	0.339*	0.249*				
Connection	0.264*	0.268*	0.279*	0.208*	0.233*			
Eating	0.201*	0.172*	0.307*	0.210*	0.238*	0.147*		
Alcohol	0.188*	0.155*	0.201*	0.165*	0.119*	0.224*	0.159*	
Stress	0.05*	0.286*	0.299*	0.128*	0.140*	0.204*	0.110*	0.078*

*p < 0.001.

Multiple Regression was used to respond to the research question 1 and assess the ability of all six pillars to predict wellbeing using the MHC Model, the Wellbeing Model (PERMA) and the Physical Health component of the PERMA Profiler. Preliminary analyses were conducted to ensure there were no violations of the assumptions of normality (residuals), linearity, multi-collinearity, and homoscedasticity. Bonferroni correlation was applied to adjust for alpha inflation. The first model accounted for 19% of variance in MHC [*R*^2^ = 0.19, *F*_(6, 1105)_ = 43.61, *p* < 0.001]. All LM pillars were statistically significant, with the three highest beta values showing for stress (β = 0.23, *p* < 0.001), social (β = 0.14, *p* < 0.001), and exercise (β = 0.12, *p* < 0.001). The second model accounted for 16% of the variance in PERMA [*R*^2^ = 0.16, *F*_(6, 1105)_ = 35.94, *p* < 0.001]. All the LM pillars were statistically significant at *p* < 0.05, with the highest beta values showing for stress (β = 0.22, *p* < 0.001), social (β = 0.16, *p* < 0.001). The third model accounted for 26% of variance in Physical Health [*R*^2^ = 0.26, *F*_(6, 1105)_ = 63.11, *p* < 0.001]. Five out of six LM pillars were statistically significant, with the highest values reported for exercise (β = 0.22, *p* < 0.001). The only pillar that was not statistically significant was sleep. See Table 3 for details.

**TABLE 3:** Multiple regression models to assess the ability of lifestyle medicine (LM) pillars to predict variance in wellbeing using mental health continuum (MHC), PERMA, and physical health.

Variables	B	SE	β	*R* ^2^	F for change
Sleep	0.16	0.05	0.09*	0.19	43.61
Exercise	0.21	0.05	0.12**		
Social	0.23	0.05	0.14**		
Eating	0.16	0.05	0.09*		
Alcohol	0.16	0.05	0.10*		
Stress	0.44	0.05	0.23**		
		**PERMA**			
Sleep	0.29	0.10	0.09*	0.16	35.94
Exercise	0.32	0.10	0.09*		
Social	0.55	0.10	0.16**		
Eating	0.26	0.10	0.08*		
Alcohol	0.22	0.10	0.07*		
Stress	0.83	0.11	0.22**		
		**Health**			
Sleep	0.07	0.13	0.02	0.26	63.11
Exercise	1.06	0.14	0.22**		
Social	0.64	0.14	0.13**		
Eating	0.94	0.13	0.20**		
Alcohol	0.45	0.13	0.10**		
Stress	1.13	0.14	0.21**		

*p < 0.05, **p < 0.001.

### The odds of using lifestyle medicine pillars

A chi-square test of independence was performed to respond to the research question 2 and 3 by examining the relation between those who are moderately well vs. flourishing and those who are languishing vs. flourishing in relation to using 0–2 vs. 3–6 LM pillars in their lives. The results showed that individuals who flourished, compared to moderately well, were more likely to use 3 or more LM pillars *X*^2^ (1, *N* = 1075) = 78.12, *p* = 0.000. Similarly, those who flourished were more likely to use 3–6 pillars compared to individuals who languished *X*^2^(1, 635) = 36.68, *p* < 0.001. Furthermore, the odds ratio model showed that individuals who were flourishing were three times more likely to use 3–6 LM pillars than those who were moderately well (OR = 3.10, 95% CI = 2.41, 3.99); and those who flourished were nine times more likely to use 3–6 LM pillars than those who languished (OR = 8.83, 95% CI = 3.96, 19.71).

## Discussion

This is the first research identifying a direct link between the six LM pillars and wellbeing, using the MHC and PERMA models of flourishing. These findings are in line with previous research that describes the positive impact of individual LM pillars on increased wellbeing (Blanchflower et al., 2013; Akbari et al., 2021; Burns and Fardfini, 2021). However to date, no research has investigated the collective impact of engaging all the LM pillars on flourishing.

Moreover, the current study identified that those who used 3–6 LM pillars were more likely to flourish. Specifically, flourishers were three times more likely than those who were moderately well and nine times more likely than languishers to use three or more LM pillars. This is in contrast with other research showing that when using psychological interventions to enhance wellbeing, the number of wellbeing activities is not necessarily correlated with more improvement in wellbeing. Individuals tend to disengage from these activities due to boredom caused by repetition (Lyubomirsky and Layous, 2013; Parks and Biswas-Diener, 2013).

The reason for the difference between the impact of LM pillars and psychological interventions on flourishing may lie in the mechanisms through which LM pillars influence wellbeing. For example, eating a varied and healthy diet with an emphasis on fruit and vegetables, is known to promote a healthy gut microbiome (Appanna, 2018). Healthy gut flora metabolize food to produce neurotransmitters (serotonin from tryptophan), as well as short chain fatty acids such as butyrate (from complex dietary carbohydrates) that can stabilize mood and stimulate the Vagus nerve (Cryan et al., 2019). Therefore, combining a healthy diet (Appanna, 2018) with exercise (Peluso and Guerra de Andrade, 2005) might have a synergistic impact on the positive regulation of neurotransmitters and endorphins, compared with either, practiced in isolation. Conversely, although we know that cultivating optimism (Lee et al., 2019) is important for health and wellbeing, it might not have the same impact as diet on our mood, for the reasons described above. Further research is required to confirm the impact of LM pillar dosage on flourishing using varied sample populations. It will be necessary to explore the intricacies of practices related to LM pillars, compared with typical PPIs, such as gratitude or savoring.

More importantly our findings offer an opportunity for researchers and practitioners to expand on existing PPIs, to address the shortage of interventions aimed at improving positive outcomes (Pawelski, 2020). Moreover, this research provides evidence that higher levels of psychological wellbeing can be accomplished by not only using psychological tools but also *via* interventions that aim to enhance physical wellbeing, such as exercise, eating well or maintaining good sleep hygiene. Future research should explore this link further, by delving deeper into the individual LM pillars as part of the model. For example, it will be important to include tobacco and other risky substances in the substance-control pillar. Likewise, LM places a strong emphasis on consuming a healthy, predominantly plant-based or vegetarian diet for reducing the risk of developing NCDs (ACLM, 2022a). It will be crucial to examine links between standard Western, vegetarian, pescatarian, flexitarian, lacto-ovo and vegan diets and flourishing. In addition, given the positive impact of healthy behaviors linked to the LM pillars, on aging (both chronological and epigenetic) (Vodovotz et al., 2020), future research should explore the links between PPI and LM in this field.

Interestingly, the LM pillar most frequently used by participants in this study to enhance their wellbeing was exercise. Exercise can have a positive influence on reducing the symptoms of affective disorders. For example, Jabakhanji et al. (2022) analyzed data from a network meta-analysis of 7,240 patients regarding the most cost-effective treatment for depression among patients in remission with coronary artery disease. They found that exercise was most effective in preventing depression when examined 8 weeks post-intervention (Jabakhanji et al., 2022). The current study provides additional evidence on the role exercise plays in helping individuals flourish, and not only reducing illness. This is important, given the scant research relating to the impact of exercise on flourishing, rather than the reduction in illness (Salama-Younes, 2011). Future research needs to examine the bidirectional nature of flourishing and exercise and delve deeper into the frequency and types of exercise, not only whether participants exercise or not.

To illustrate this point, the Biopsychosocial Religion and Health (BRH) Study of Seventh-Day Adventists is a prospective longitudinal study that examined health data (approximately 96,000 North American participants and members of the Seventh-Day Adventist religion) between 2006 and 2011 (Lee et al., 2009). After assessing 5,789 participants from this BRH study, the researchers found a bidirectional relationship between exercise and flourishing, specifically related to the ratio of positive to negative affect (Leibow et al., 2021).

Finally, this study may contribute to research exploring the link between positive mental health and healthy lifestyle practices in healthcare, counseling and coaching contexts. For example, the Make Every Contact Count (MECC) initiative in Ireland (HSE, 2022) and the United Kingdom (Lawrence et al., 2016) involves training healthcare professionals to use every engagement with service users as an opportunity to support behavior changes that can contribute toward healthier lifestyles. This approach involves social prescribing (Husk et al., 2019) and the provision of important information on the pillars of LM as they contribute to health. MECC is especially important for patients suffering from chronic diseases and the prevention of these diseases in otherwise healthy service users. Evidence-based information regarding the positive connection between the pillars of LM and flourishing is very useful for healthcare workers employing MECC in the clinic or the community. Likewise, it is essential for mental health workers, counselors, psychotherapists and coaches to access empirical data describing the positive relationship between human flourishing and everyday lifestyle practices. Whole person health, which incorporates the Salutogenic model (Antonovsky, 1996), is now widely viewed as integrating mental and physical wellbeing with positive social and environmental health (Kligler, 2022). The findings of this study will add to the increasing body of evidence highlighting the links between positive mental health and healthy lifestyle practices.

## Limitations

Despite the significant benefits and novel findings, the current research has three main limitations. Firstly, this homogenous sample included only professionals, the majority of whom were female, aged 40 plus. Participants were members of a specific cohort of leaders in education, which may have influenced the results. Further research should consider sampling a general and more diverse population. Secondly, past research showed that on average, between 10 and 15% of the population experiences languishing at any given time (Keyes, 2002). In the current sample, only 3% of participants reported languishing, meaning that the current sample has an unusually low prevalence of risk for mental health issues. The reason for it may be due to participants’ profession or age. Future research will include a more diverse and general sample of population, including non-leaders. Finally, the questions used to assess participation in LM pillars require expansion and development. Further research will include more concise and specific questions related to each pillar. These questions will expand on the multiple nuances associated with LM pillars and not simply include questions that require dichotomous yes/no answers. For example, we will expand questions on physical activity to include the type of activity, duration and intensity. The lack of a validated instrument that measures the extent of engagement with all LM pillars is a problem that requires urgent attention.

## Conclusion

The current research provides preliminary evidence showing that psychological flourishing can be predicted by examining the level of engagement with the six pillars of LM. Furthermore, it demonstrated that flourishers were three times more likely to use 3–6 LM pillars than individuals who reported moderate wellbeing; moreover, they were nine time more likely to use 3–6 LM pillars than those who languished. This novel finding opens the door to using LM pillar-based interventions as an alternative to, or in conjunction with PPIs, to cultivate flourishing.

These findings can be used by researchers to further explore the link between LM and flourishing, especially by expanding on the behaviors associated with each LM pillar. We hope that this research will encourage professionals (e.g., healthcare staff, counselors, and coaches) to help clients engage the LM pillars, to not only improve their physical health and reduce illness, but also to enhance their psychological wellbeing. Finally, policymakers are encouraged to consider and incorporate LM pillars as part of the guidelines for improving public health and wellbeing.

## Data availability statement

The data analyzed in this study is subject to the following licenses/restrictions: no restrictions. Data is available upon request. Requests to access these datasets should be directed to JB.

## Ethics statement

The studies involving human participants were reviewed and approved by the Maynooth University. The patients/participants provided their written informed consent to participate in this study.

## Author contributions

JB collected the data. JB and PD co-wrote a manuscript. Both authors contributed to the article and approved the submitted version.
